# From Childhood Migraine Headache to Pheochromocytoma

**DOI:** 10.1155/2014/746723

**Published:** 2014-05-25

**Authors:** Y. M. Hazimeh, M. Luidens, M. E. Ehlers, V. Sharma

**Affiliations:** Endocrinology Department, South Clinical Campus, Albany Medical College, Albany, NY 12208, USA

## Abstract

Pheochromocytoma may have multiple clinical manifestations including paroxysmal hypertension, tachycardia, sweating, nausea, and headache (Phillips et al., 2002). Migraine has some of the manifestations seen with pheochromocytoma. We describe a patient who had a history of migraine headaches since childhood and was found to have pheochromocytoma. Resection of her tumor significantly improved her headache. The diagnoses of pheochromocytoma subsequently lead to diagnosing her with medullary thyroid cancer (MTC) and multiple endocrine neoplasia type 2A (MEN-2A).

## 1. Introduction

Pheochromocytomas are rare tumors [1], and headache is one of their most common symptoms [2]. MEN-2A is an autosomal dominant disorder that causes familial pheochromocytomas and is associated with MTC and parathyroid adenoma [3]. Migraine headache is not well prevalent in children [4]. We present a 20-year-old female with severe migraine since childhood and no family history of pheochromocytoma. She had an incidental adrenal mass that turned out to be pheochromocytoma. Further work-up revealed MTC and MEN-2A.

## 2. Clinical Case

**Figure 1:**
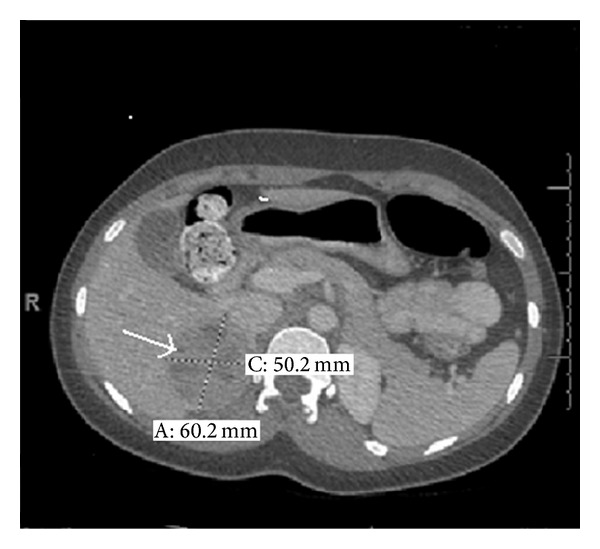
There is a heterogeneous mass measuring 6.0 × 5.0 × 4.9 cm in the expected location of right adrenal gland (arrow).

**Figure 2:**
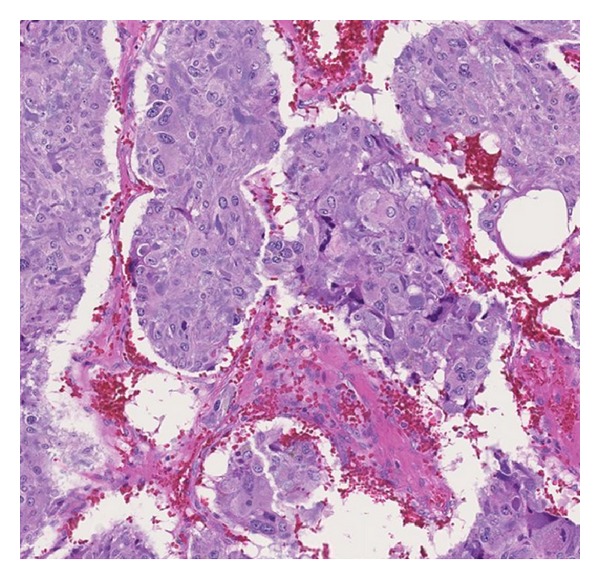
Pheochromocytoma: nests and sheets of amphophilic polygonal cells are separated by vascular spaces.

A 20-year-old female, with a history of migraine since the age of seven, presented to the emergency department with headache, abdominal pain, nausea, and vomiting. She has been on topiramate, cyproheptadine, Fioricet, oxycodone, and sumatriptan for migraine headaches for the previous six years. She was seen in a different emergency room two days before for the same reason and was diagnosed with a viral illness. She described her abdominal pain on arrival as severe. Her blood pressure in the emergency department was 130/80 and heart rate 112 BPM. She was afebrile. Her abdomen was slightly tender to palpation without any palpable mass. Physical exam was otherwise unremarkable. She had an elevated WBC of 10900 cells/mcl with 79% segmented. CT scan of abdomen and pelvis was done to rule out missed appendicitis. CT was negative for intra-abdominal infection; however, it revealed a heterogeneous 6.0 cm right adrenal mass (Figure 1). She was admitted to the hospital. On the second day, she had an elevated blood pressure of 210/100 mmHg. This was associated with headache and tachycardia of 110 BPM. She had a work-up for the adrenal mass including 24-hour urine collection for cortisol and catecholamines. Results showed a cortisol of 78 mcg/24 hr (normal (nl) < 50), metanephrine of 1,195 mcg/24 hr (nl 24–290), normetanephrine of 6,680 mcg/24 hr (nl 82–500), dopamine of 244 mcg/24 hr (nl 65–400), epinephrine of 50 mcg/24 hr (nl 0–20), and normetanephrine of 815 mcg/24 hr (nl 0–135) with creatinine level indicating adequate collection. Serum aldosterone was 2.6 ng/dL (nl 1–16), renin 49 ng/dL/hr (nl 20–160), and chromogranin A 116 (nl < 15). Her serum electrolytes and complete blood count were normal. Her blood pressure was treated adequately with phenoxybenzamine followed by metoprolol. One week later she underwent right adrenalectomy and her blood pressure normalized without any medication. Pathology was consistent with pheochromocytoma (Figure 2). After subsequent follow-up, her migraine attacks became less frequent and she stopped three of her pain medications. Serum calcitonin level checked in the follow-up visit was 30.1 pg/mL (nl < 15). Genetic testing came back positive for RET germline mutation involving codon 634. She was referred to thyroidectomy and was found to have medullary thyroid carcinoma.

## 3. Discussion 

Pheochromocytomas are rare tumors with an annual incidence of 0.8 cases in 100,000 person-years [1]. These tumors can cause paroxysmal hypertension in approximately 50% of cases [5]. This could explain why our patient was not previously diagnosed with hypertension despite routine follow-up with her primary care physician and neurologist. Headache occurs in up to 90% of pheochromocytoma cases [2]. The chronicity of this patient's headache suggests that her pheochromocytoma has been present since childhood. Her migraine has been severe since the age of thirteen and required five medications to control adequately. Migraine affects 2–7% of children [4]. In the United States, during one year, approximately 20 percent of children aged 4 to 18 years report having had frequent or severe headaches [6]. Looking for a pheochromocytoma in all of these cases would not be cost-effective.

Most pheochromocytomas are sporadic and approximately 10% are familial [3]. In familial cases of pheochromocytoma, the disease is inherited as autosomal dominant [7]. This patient has no previous family history of pheochromocytoma that might have prompted screening her earlier for the disease. In sporadic cases of pheochromocytoma, up to 25 percent will have unsuspected germline mutations of the RET, VHL, SDHD, SDHB, SHDC, SDHAF2, SDHA, TMEM127, or MAX gene [8]. Genetic testing of patients with sporadic pheochromocytoma may identify other family members at risk for associated tumors. Finding a germline mutation will affect further management of patients. In this case, the patient was found to have a mutation in RET protooncogene. This mutation is associated with multiple endocrine neoplasia type 2A [9]. The mutation was in the codon 634 leading to a substitution of amino acid cysteine to arginine (C634R) in the exon 11 of RET gene. Her calcitonin level was elevated and she was referred to thyroidectomy. She was found to have medullary thyroid carcinoma. Due to potentially aggressive MTC associated with C634R mutation, American Thyroid Association recommends prophylactic thyroidectomy before the age of five for patients known to be carrier of this mutation [9]. While the majority of patients carrying the C634R mutation may develop MTC and pheochromocytoma, only a minority develops primary hyperparathyroidism [9]. This patient's calcium and parathyroid hormone levels were in the normal range.

## 4. Conclusion

Headache is a highly prevalent symptom of pheochromocytoma. Children and adolescents with long-term headache, requiring multiple medications, may need to be screened for missed paroxysmal hypertension and subsequently pheochromocytoma. Diagnosing pheochromocytoma in this age group may have significant impact on managing headaches, as well as on long-term complications and survival. It may also have implications for other family members.
